# Multiple myeloma presenting as bilateral orbital proptosis

**DOI:** 10.4103/0301-4738.55069

**Published:** 2009

**Authors:** Archana Malik, Subina Narang, Uma Handa, Sunandan Sood

**Affiliations:** Department of Ophthalmology, Government Medical College and Hospital, Chandigarh - 160 032, India; 1 Department of Pathology, Government Medical College and Hospital, Chandigarh - 160 032, India

**Keywords:** Bilateral proptosis, multiple myeloma, plasma cell dyscrasias.

## Abstract

A 58-year-old-man presented with painful rapidly progressive bilateral proptosis with restricted ocular movements of 15 days duration. There was history of significant weight loss in the recent past. Computed tomography scan of the head and orbit revealed bilateral multiple, well-defined, round, soft tissue masses, isointense with muscles in intraconal and extraconal space. Fine needle aspiration cytology and incision biopsy from the lesion, urine for Bence-Jones proteins and immunofixation clinched the diagnosis of multiple myeloma. Skeletal survey did not reveal any bony involvement.

The diagnosis of multiple myeloma should be kept in mind in cases of bilateral proptosis. Bony involvement is not universal in cases of orbital myeloma. Early diagnosis can be established with extensive biochemical and histopathological investigations and timely treatment is life saving for these patients.

Multiple myeloma is a subgroup of plasma cell dyscrasias in which there is neoplastic proliferation of plasma cells or their precursors. Unilateral proptosis is a well-recognized but somewhat rare presentation of multiple myeloma in ophthalmic practice.[1,2] Bilateral proptosis with multiple soft tissue masses is an extremely rare presentation of multiple myeloma.[3] We report a case of multiple myeloma that presented with bilateral proptosis due to multiple orbital soft tissue lesions without any bony involvement.

## Case Report

**Figure 1:**
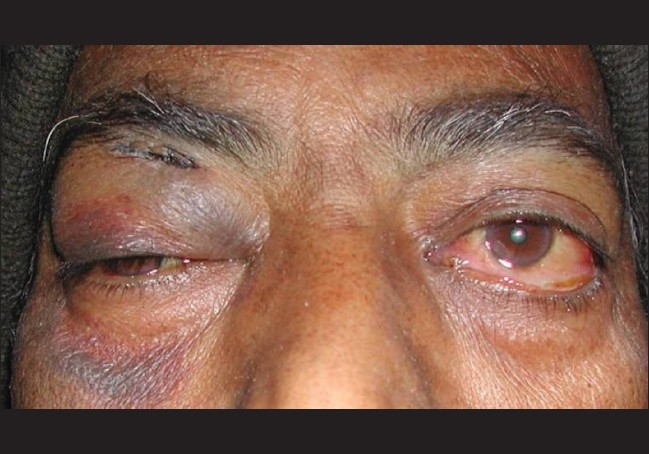
Photograph showing proptosis with conjunctival chemosis. Right eye periorbital ecchymosis is post fine needle aspiration cytology

**Figure 2:**
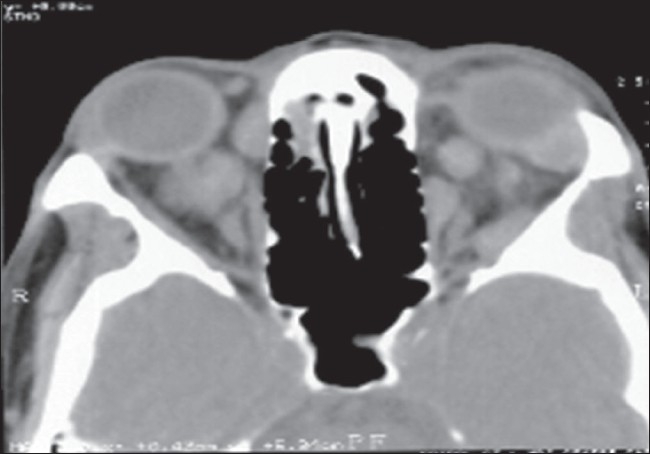
Computer tomography scan of orbit revealed bilateral multiple, well-defined, round, soft tissue masses, isointense with muscles in intraconal and extraconal space without any bony erosion

A 58-year-old-man presented with painful rapidly progressive bilateral proptosis with restricted ocular movements of 15 days duration. There was history of about 10-kg weight loss amounting to 15% of his body weight over a period of the past one month. Ophthalmic examination revealed visual acuity of 20/30 in both eyes. His right eyeball was pushed inferiorly and laterally and left eye showed axial proptosis [Fig. 1]. Hertel's exophthalmometry reading was 24 mm in the right eye (RE) and 21 mm in the left eye (LE) with intercanthal distance of 110 mm. RE was shifted inferiorly by 5 mm and laterally by 2 mm as measured with plastic scale. There was increased retrobulbar resistance. In the RE, maximal restriction of ocular movements was noted in levoelevation and adduction with mild restriction in other gazes. There was mild restriction of movements in all gazes in the LE. On deep palpation a smooth, round, firm, non-tender mass (approximately 2.5 cm × 2 cm) could be palpated in the superior orbit of the RE. The posterior extent of the mass could not be reached and it was free from the overlying skin. No mass lesion could be palpated in the LE. The proptosis was non-compressible and did not have any postural variation. Anterior segment biomicroscopy and pupillary reactions were normal in both eyes except for higher intraocular pressure in the RE of 24 mm of Hg on Goldman applanation tonometry. The intraocular pressure was 12 mm of Hg in the LE. Fundus examination revealed few scattered hemorrhages and cotton-wool spots in both eyes. Computed tomography (CT) scan of the head and orbit revealed bilateral multiple, well-defined, round, soft tissue masses, isointense with muscles in the intraconal and extraconal space [Fig. 2]. Three days after his initial presentation to us, the patient noticed development of subcutaneous nodules on his left thigh and abdomen.

The differential diagnosis of secondaries from an unknown primary, blood cell-related tumor including lymphoma or plasma cell dyscrasias was kept. Hemogram and coagulation profile showed hemoglobin of 7.8 g/dl (Normal: 14-16 g/dl) though his blood counts and coagulation profile was normal. To look for an unknown primary from the lung, prostate, gastrointestinal tract; endoscopy, colonoscopy, stool examination and prostate examination was done. Examination of the gastrointestinal tract (GIT) and prostate was unremarkable. CT abdomen revealed paravertebral soft tissue mass and contrast enhanced CT (CECT) chest revealed a soft tissue mass in the parahilar region.

**Figure 3:**
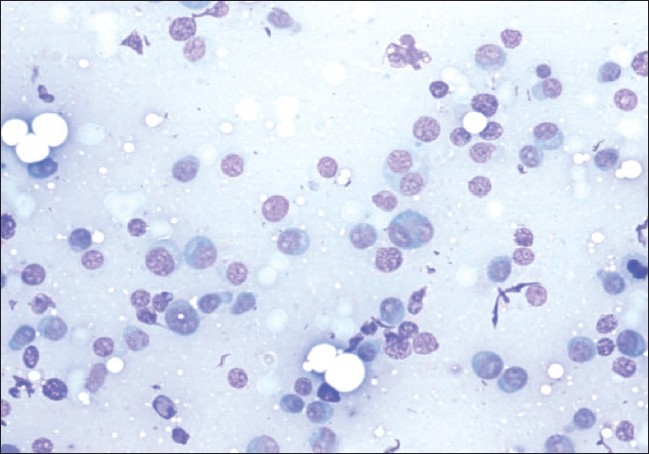
Fine needle aspiration biopsy of orbital mass showing cellular sheets of mature and immature plasma cells, many bi-nucleate and few multinucleate forms on May Grunwald Giemsa stain (200x)

**Figure 4:**
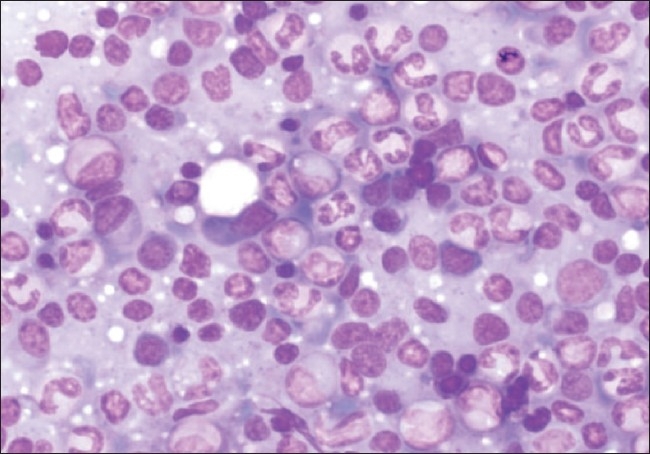
Hypercellular bone marrow smear shows predominance of mature plasma cells along with few binucleate forms and plasmacytoid lymphocytes on May Grunwald Giemsa stain (200x)

Fine needle aspiration cytology (FNAC) followed by incision biopsy of the orbital mass and FNAC from the left thigh nodule showed cellular sheets of mature and immature plasma cells, many binucleate and multinucleate forms were seen, suggestive of plasmacytoma [Fig. 3]. Keeping in the mind diagnosis of multiple myeloma Bence-Jones protein testing, bone marrow examination, skeletal survey and immunofixation was done. The urine was positive for Bence-Jones protein. Serum and urine electrophoresis showed a sharp M-band in the gamma region. Immunofixation confirmed the diagnosis of IgG multiple myeloma. Skeletal survey, however, did not reveal any lytic lesions. Bone marrow examination subsequently revealed >20% plasma cells and >36% lymphoplasmacytic cells [Fig. 4]. Renal function tests (RFT) and serum electrolytes were done as their derangement is common in multiple myeloma. The RFT (Normal: blood urea- 15-45 mg/dl, serum creatinine-0.8-1.8 mg/dl) were deranged 10 days after admission with blood urea 64 mg/dl, serum creatinine 3.1 mg /dl.

**Figure 5:**
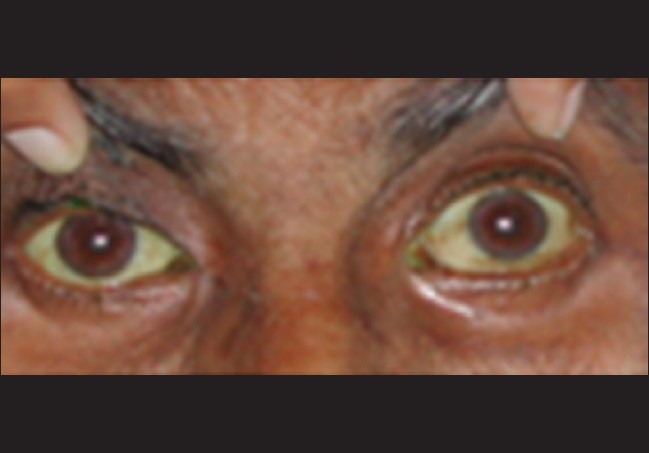
Resolution of proptosis three weeks after initiation of chemotherapy

Patient was started on chemotherapy in consultation with the oncologist (Pulse therapy of Melphalan and Prednisolone). Patient responded dramatically to treatment with remission of eye manifestations [Fig. 5] and skin nodules within three weeks of starting treatment.

## Discussion

Plasma cell dyscrasias are a rare cause of proptosis. Solitary extramedullary plasmacytoma (SEMP) tends to be locally invasive and does not often metastasize. Multiple myeloma is a malignant systemic plasma cell neoplasm that is aggressive and metastasizes. It is a malignancy of plasma cells characterized by monoclonal proliferation of a single clone of highly specialized B-lymphocytes engaged in the production of a single immunoglobulin. It is associated with production of paraproteins and/or light chain moiety. The criteria for diagnosis of SEMP include negative lymph node assessment, skeletal survey, bone marrow biopsy and CT. In the present case the patient though presented with proptosis and no skeletal involvement, had positive bone marrow biopsy, renal insufficiency, anemia and multiple plasmacytomas. Orbital involvement is a rare presentation of the disease.[1,2,4] Unilateral proptosis is the most common form of orbital involvement. On computerized Medline search we found only five case reports of bilateral proptosis in multiple myeloma[3,5–8] and only seven previous cases of cytological diagnosis.[4]

Orbital myeloma most commonly presents as a unilateral solitary soft tissue intraorbital tumor which is an extension of bony deposit and is associated with bone destruction.[9] In the present case multiple well-defined soft tissue tumors were not associated with bony lesions. In all previously described orbital multiple myeloma cases, soft tissue masses were seen to infiltrate the globe or muscles and skeletal survey showed radiolucent areas on skull.[3,5–8] In our case there was also presence of small paraspinal soft tissue masses without bony involvement, and radiological features simulated secondaries and lymphomas. The diagnosis could only be established after bone marrow and biochemical investigations in the present case. Histopathological picture was suggestive of multiple myeloma and immunofixation confirmed the diagnosis of IgG multiple myeloma. It is essential to rule out systemic involvement by intensive workup in cases presenting as a solitary orbital plasmacytoma as the treatment and prognosis differs with systemic involvement.[10,11]

The diagnosis of multiple myeloma should be kept in mind in cases of bilateral proptosis. Bony involvement is not universal in cases of orbital myeloma. Early diagnosis can be established with extensive biochemical and histopathological investigations and timely treatment is lifesaving for these patients.
